# fPoxDB: fungal peroxidase database for comparative genomics

**DOI:** 10.1186/1471-2180-14-117

**Published:** 2014-05-08

**Authors:** Jaeyoung Choi, Nicolas Détry, Ki-Tae Kim, Fred O Asiegbu, Jari PT Valkonen, Yong-Hwan Lee

**Affiliations:** 1 Fungal Bioinformatics Laboratory and Department of Agricultural Biotechnology, Seoul National University, Seoul 151-921, Korea; 2 Center for Fungal Pathogenesis, Seoul National University, Seoul 151-921, Korea; 3 Department of Forest Sciences, University of Helsinki, 00014 Helsinki, Finland; 4 Department of Agricultural Sciences, University of Helsinki, 00014 Helsinki, Finland; 5 Center for Fungal Genetic Resources, Plant Genomics and Breeding Institute, and Research Institute for Agriculture and Life Sciences, Seoul National University, Seoul 151-921, Korea

## Abstract

**Background:**

Peroxidases are a group of oxidoreductases which mediate electron transfer from hydrogen peroxide (H_2_O_2_) and organic peroxide to various electron acceptors. They possess a broad spectrum of impact on industry and fungal biology. There are numerous industrial applications using peroxidases, such as to catalyse highly reactive pollutants and to breakdown lignin for recycling of carbon sources. Moreover, genes encoding peroxidases play important roles in fungal pathogenicity in both humans and plants. For better understanding of fungal peroxidases at the genome-level, a novel genomics platform is required. To this end, Fungal Peroxidase Database (fPoxDB; http://peroxidase.riceblast.snu.ac.kr/) has been developed to provide such a genomics platform for this important gene family.

**Description:**

In order to identify and classify fungal peroxidases, 24 sequence profiles were built and applied on 331 genomes including 216 from fungi and Oomycetes. In addition, NoxR, which is known to regulate NADPH oxidases (NoxA and NoxB) in fungi, was also added to the pipeline. Collectively, 6,113 genes were predicted to encode 25 gene families, presenting well-separated distribution along the taxonomy. For instance, the genes encoding lignin peroxidase, manganese peroxidase, and versatile peroxidase were concentrated in the rot-causing basidiomycetes, reflecting their ligninolytic capability. As a genomics platform, fPoxDB provides diverse analysis resources, such as gene family predictions based on fungal sequence profiles, pre-computed results of eight bioinformatics programs, similarity search tools, a multiple sequence alignment tool, domain analysis functions, and taxonomic distribution summary, some of which are not available in the previously developed peroxidase resource. In addition, fPoxDB is interconnected with other family web systems, providing extended analysis opportunities.

**Conclusions:**

fPoxDB is a fungi-oriented genomics platform for peroxidases. The sequence-based prediction and diverse analysis toolkits with easy-to-follow web interface offer a useful workbench to study comparative and evolutionary genomics of peroxidases in fungi.

## Background

Peroxidases (EC 1.11.1.x) are a group of oxidoreductases that catalyse the oxidation of various compounds by using peroxides. While hydrogen peroxide (H_2_O_2_) is commonly used as an electron donor, peroxidases can take a variety of different substrates as electron acceptors. Peroxidases can be divided into two major groups, contingent upon the presence or absence of a haem cofactor. Among their numerous industrial applications, one good example would be their ability to remove phenolic compounds from wastewater, in which haem peroxidases are involved. For instance, peroxidases including horseradish peroxidase enzymatically catalyse the conversion of phenolic substrates into phenoxy radicals. The resulted phenoxy radicals can chemically react among themselves or with other substrates, consequently causing precipitation of polymeric products, which can be easily separated from the wastewater [1,2]. In addition, lignin peroxidase (LiP) and manganese peroxidase (MnP) are considered to be the most effective enzymes for recycling carbon sources fixed as lignin [3]. As genes encoding LiP are quite limited to white rot fungi, including *Phanerochaete chrysosporium*[4,5], *P. sordida*[6], *Trametes versicolor*[7], *Phlebia radiata*[8,9], *P. tremellosa*[10], and *Bjerkandera* sp. [11], genes encoding MnP have drawn attention as an alternative ligninolytic peroxidase due to their wider distribution among basidiomycetes compared to those encoding LiP. Furthermore, site-directed mutagenesis on LiP and MnP genes revealed that the catalytic residues play pivotal roles in switching enzymatic activities between LiP and MnP in *P. chrysosporium*[12,13]. Recently, a new type of haem protein called versatile peroxidases (VPs) has been found in *Pleurotus* and *Bjerkandera* species that can naturally perform both functions [14,15]. Hence, they are considered to be another candidates for ligninolysis. Meanwhile, a dye-decolorizing peroxidase (DyP), MsP1, in *Marasmius scorodonius* is thought to be useful for industrial applications due to its high temperature and pressure stability [16]. Besides their industrial impacts, peroxidases are also important in fungal pathogenicity on host animals and plants. For example, deletion mutants of a gene encoding thiol peroxidase, *TSA1*, in *Cryptococcus neoformans* showed significantly less virulence on mice [17]. For plant pathogens, peroxidases are required to detoxify host-driven reactive oxygen species for *Ustilago maydis*[18] and *Magnaporthe oryzae*[19]. In addition, mutants of genes encoding NADPH oxidases (Nox) in *Botrytis cinerea*, *bcnoxA* and *bcnoxB*, showed attenuated virulence on citrus where double knockout or deletion of the gene encoding regulatory protein, *bcnoxR*, gave additive effects [20].

Along with the industrial and biological importance of peroxidases, together with the availability of fully sequenced fungal genomes, a genomics resource is required for better understanding of peroxidases at the genome-level. Peroxidase genes might be identified by using domain prediction tools, such as InterPro scan [21] or Pfam [22]. However, identification based on domain profiles could result in false positives. For example, NoxA [23] and a metalloreductase (FREA) [24] in *Aspergillus nidulans* showed the same domain profiles predicted by InterPro scan [21] and Pfam [22]. Since ferric reductases (FRE) and ferric-chelate reductases (FRO) share high structural similarity with Nox [25], the gene encoding FREA would become a false positive in domain-based prediction of Nox genes. Because filtering out false positives is an important issue in studying comparative or evolutionary genomics on Nox genes, Nox family is divided into three subfamilies, NoxA, NoxB, and NoxC. Previously, a database named as PeroxiBase [26] was developed to archive the genes encoding peroxidases in a wide range of taxonomy. Although PeroxiBase contains fungal peroxidases, it does not specifically focus on fungi and archive genes encoding NoxR, which are known to regulate NoxA and NoxB in fungi [27-29]. Hence, it is necessary to build a peroxidase database for comparative and evolutionary analysis in fungi.

Here, we developed a new web-based fungal peroxidase database (fPoxDB; http://peroxidase.riceblast.snu.ac.kr/) to provide a fungi-oriented archive with manually improved catalogue of Nox genes and to support comparative and evolutionary genomics of genes encoding various peroxidases. Finally, we show an overview of the taxonomic distribution of peroxidase genes in the kingdom Fungi which could be applied for investigation of phylogenetic relationship.

## Construction and content

### Construction of the pipeline for identification of the genes encoding peroxidases

**Figure 1:**
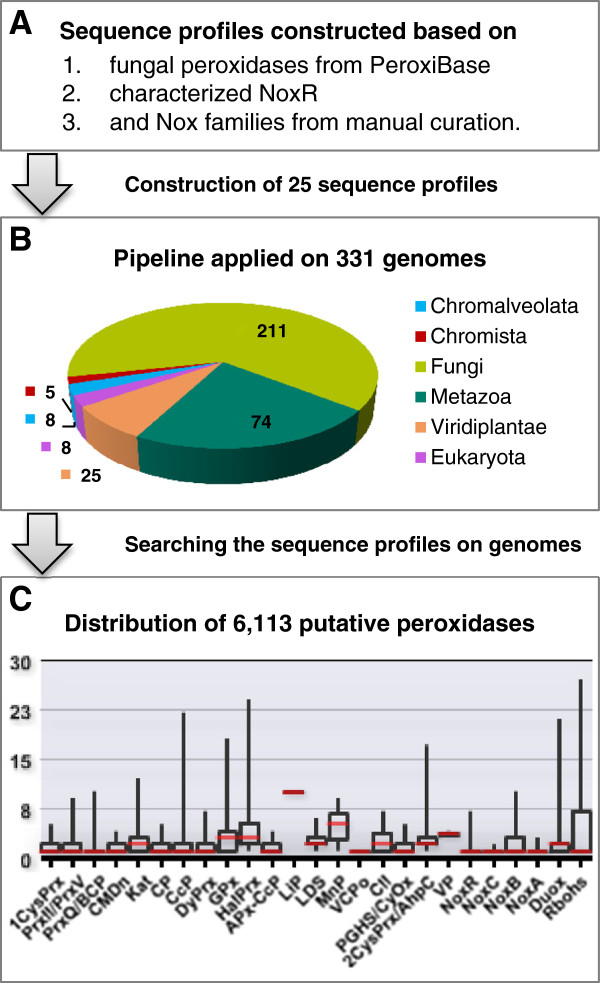
**The pipeline and contents of fPoxDB.** A schematic diagram of fPoxDB pipeline and contents. A computational prediction pipeline is composed of preparation of raw sequences **(A)**, searching 331 target genomes with 25 sequence profiles **(B)** and 6,113 predicted genes as the end product **(C)**. The median value for each gene family is indicated by a red line **(C)**.

**Figure 2:**
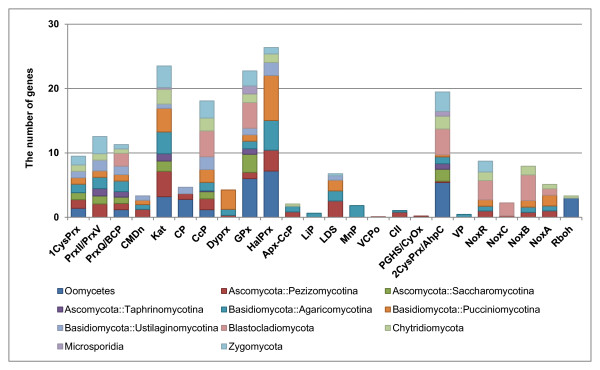
**Taxonomic distribution of gene families.** The average numbers of putative genes for each peroxidase family are plotted against the subphylum-level of taxonomy in fungi and Oomycetes.

In order to set up a pipeline for fPoxDB, the protein sequences of fungal peroxidases were retrieved from PeroxiBase [26]. Particularly, the gene family “Ancestral NADPH oxidase” was redefined with three gene families, NoxA, NoxB, and NoxC. Protein sequences of two other NADPH oxidase families, Duox (dual oxidase), and Rboh (respiratory burst oxidase homologue), were also included. Majority of Duox and Rboh were found in animals and plants, respectively. They were integrated into fPoxDB to detect their remote homologues in fungi. In addition, protein sequences of NoxR, the regulatory subunit of NoxA and NoxB, were collected from various literatures. The protein sequences for each gene family were subjected to multiple sequence alignment by using T-Coffee [30], then manually curated and trimmed for refinement. The refined alignment for each gene family was used as an input for the construction of a sequence profile, which was done by *hmmbuild* in the HMMER package (version 2.3.2) [31]. The resulting sequence profiles were searched on 331 genomes, which were obtained from the standardized genome warehouse of Comparative Fungal Genomics Platform (CFGP 2.0; http://cfgp.snu.ac.kr/) [32], to find putative genes encoding peroxidases (Figure 1). As a result, 6,113 peroxidase genes were predicted from 331 genomes including 216 from fungi and Oomycetes (Table 1, Figure 1, and Additional file 1). As expected, peroxidase genes were found in every taxon, implying its essentiality in fungal physiology and metabolism. However, the average number of peroxidase genes per genome was turned out to be different between Ascomycota (15.66) and Basidiomycota (23.95), and among the three subphyla in Ascomycota. On average, the species in Basidiomycota had more peroxidase genes than the ones in Ascomycota (t-Test; *P* = 5.0e^-3^). Within Ascomycota, the three major subphyla Pezizomycotina, Saccharomycotina, and Taphrinomycotina had the average gene number of 24.29, 10.69, and 4.97, respectively, with significant differences (t-Test; *P* ≤ 1.2e^-21^). However, no significant differences were observed among the species in Basidiomycota. On the other hand, Oomycetes were predicted to have 31.40 peroxidase genes, on average. Interestingly, though the average number of genes in Oomycete genomes was larger than those in fungi (16.36) (t-Test; *P* = 5.0e^-4^), the predicted genes were found in fewer gene families (8.4 per genome, on average) than those belonging to the subphyla Pezizomycotina (13.60) and Agaricomycotina (12.31), but more than those of Saccharomycotina (6.93) and Taphrinomycotina (4.57) (Figure 2 and Additional file 1).

**Table 1:** Summary of peroxidase families found in fungal and Oomycete genomes

**Category**	Gene family^*^	**Number of genes**	**Number of genomes**
Haem peroxidase	Catalase	542	209
	Catalase-peroxidase	76	49
	Cytochrome C peroxidase	285	203
	DyP-type peroxidase D	45	24
	Haloperoxidase	364	75
	Hybrid Ascorbate-Cytochrome C peroxidase	73	48
	Lignin peroxidase	10	1
	Linoleate diol synthase (PGHS like)	206	81
	Manganese peroxidase	29	6
	NADPH oxidase, NoxA	89	84
	NADPH oxidase, NoxB	77	70
	NADPH oxidase, NoxC	18	17
	NADPH oxidase, Duox^**^	0	0
	NADPH oxidase, Rboh^***^	16	5
	Other class II peroxidase	58	22
	Prostaglandin H synthase (Cyclooxygenase)	13	13
	Versatile peroxidase	7	2
Non-haem peroxidase	1-Cysteine peroxiredoxin	245	200
	Atypical 2-Cysteine peroxiredoxin (typeII, typeV)	325	205
	Atypical 2-Cysteine peroxiredoxin (typeQ, BCP)	218	200
	Carboxymuconolactone decarboxylase	100	73
	Fungi-Bacteria glutathione peroxidase	437	210
	No haem, Vanadium chloroperoxidase	5	5
	Typical 2-Cysteine peroxiredoxin	278	151
Regulator	NoxR	93	87

^*^The gene family names were inherited from the PeroxiBase [26] that contain fungal sequences.

^**^The genes encoding Duox family were exclusively found in the species belonging to the kingdom Metazoa and *Proterospongia* sp. ATCC 50818 which belongs to the order Choanoflagellida, a close relative to the animals [33].

^***^Only one gene belonging to the Rboh family was found in fungi (*Spizellomyces punctatus*) while others were found in Oomycetes.

Six peroxidase families including 1-Cysteine peroxiredoxin, atypical 2-Cysteine peroxiredoxin (typeII, typeV), atypical 2-Cysteine peroxiredoxin (typeQ, BCP), catalase, cytochrome C peroxidase, and Fungi-Bacteria glutathione peroxidase were found in at least 200 fungal and Oomycete genomes. Particularly, species belonging to the subphyla Saccharomycotina and Taphrinomycotina had only two haem peroxidase families, but had five and four non-haem peroxidases, respectively (Additional file 1). This result might imply that the non-haem peroxidases were horizontally transferred to fungi from bacteria before diversification as they are shown to be constrained in bacteria [34]. In addition, horizontal gene transfer of haem catalase-peroxidase genes of fungi from bacteria has been reported in several previous studies [35-37]. Further study would provide better speculation on the origin of non-haem peroxidase of fungi. Surprisingly, a few gene families were limited to a certain taxon, implying their specific roles in different fungal life styles. For example, lignin peroxidase (LiP) and manganese peroxidase (MnP) were only found in the subphylum Agaricomycotina. *Phanerochaete chrysoporium* was the only species which possess the genes encoding LiP in fPoxDB. On the other hand, MnP was found in multiple species belonging to the subphylum Agaricomycotina, particularly in rot fungi including *Phanerochaete chrysosporium*, *Pleurotus ostreatus* PC9, *Dichomitus squalens*, and *Heterobasidion irregulare* TC 32–1 (Additional file 1). This is in agreement with the previous findings that these enzymes are critical in oxidation and degradation of lignin and lignocellulose [38]. According to Fungal Secretome Database (FSD; http://fsd.snu.ac.kr/) [39], all 10 LiPs and 26 MnPs belonging to these rot fungi were predicted to be secretory, which strongly supports the importance of their roles at the interface between fungal and host cells.

### Evaluation of the pipeline

In order to evaluate the prediction accuracy, 77 protein sequences annotated as peroxidase gene families were downloaded from the UniProtKB/SwissProt database [40] which was used as a positive set. In addition, to test the discrimination power against other oxidoreductase sequences, expert-curated fungal protein sequences of 39 laccases and 197 other oxidoreductases were also downloaded from the UniProtKB/SwissProt database [40] for a negative set. Laccases and other oxidoreductases are good negative sets, since these enzymes and peroxidases share the same nature in transferring electrons from one to another but take different electron donors and acceptors. As a result, all 77 protein sequences belonging to eight peroxidase families were correctly predicted by the corresponding sequence profiles in our pipeline. Furthermore, none of the 236 protein sequences from the negative set showed any significant hits. In fact, many sequences in the negative set showed insignificant hits which had far higher E-values than the identification threshold 1.0e^-5^. These results clearly supported the quality of the pipeline in the accuracy and discrimination power against the positive and negative sets, respectively.

### System architecture

fPoxDB is built on a three-tiered system which consists of database, application, and user interface tiers. The database tier embraces database servers which run on MySQL relational database management system. The application tier is comprised of system monitoring servers and computing nodes which coordinates and schedules BLAST [41], HMMER [31], BLASTMatrix [32], ClustalW [42], and analysis jobs submitted from the website. The user interface tier adopts data-driven user interface (DUI), originally designed for the CFGP 2.0 [32], which runs on the Apache HTTP Server. Servers for each tier are physically separated to balance load, providing comfortable user experience of fPoxDB. In-house scripts for the identification pipeline were written in Perl. The web interface follows HTML5 and CSS3 standard to support cross-browsing.

### Example of the database usage

Investigation of gene duplication and loss could help us to understand how fungi adapt to different environments. Catalases are haem peroxidases in which structure is well conserved throughout all domains of life [37]. They have been phylogenetically studied in both prokaryotes and eukaryotes [37,43], however, not in detail for fungi. To demonstrate how fPoxDB could be used in comparative and evolutionary studies, amino acid sequences of a domain commonly found in 109 catalases from 32 species were analysed. To elucidate evolutionary history of catalases, a reconciliation analysis was conducted. The reconciled tree revealed that duplication or loss events of catalase genes occurred frequently in most of the internal and leaf nodes (Additional file 2). Except for three nodes, all internal nodes underwent multiple gene losses or duplications in fungal clades. Interestingly, only gene losses occurred in members of Ascomycota at the species-level. In contrast, gene losses as well as duplications were found to have occurred in species belonging to Basidiomycota. The fact that basidiomycetes possess more peroxidase genes than ascomycetes suggests that the genes have evolved to adapt to their wood-decaying lifestyle. They require a large amount of catalase activity to reduce high concentration of reactive oxygen species involved in the wood decay [44]. Comparative and evolutionary analysis, such as the above-mentioned example, can be done on other families of peroxidases as well.

## Utility and discussion

**Figure 3:**
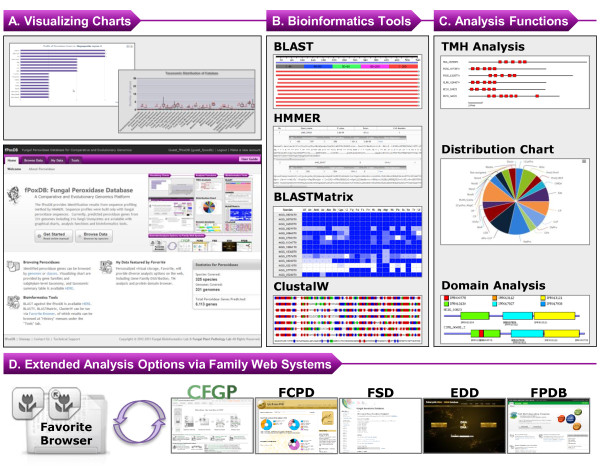
**Web interface and functionalities. A)** Web interface of fPoxDB displays well organized graphical charts for better recognition of the distribution of the genes. **B)** Tools including similarity search (BLAST [41], HMMER [31] and BLASTMatrix [32]) and multiple sequence alignment (ClustalW [42]) are provided via the Favorite Browser. **C)** Protein domain analysis and TMH analysis can be also done with the sequences collected in Favorites. **D)** Users’ sequence collection can be further analysed by the tools available at the CFGP 2.0 [32] and other sister databases [39,52-54].

The web interface of fPoxDB provides an easy-to-use genomics environment. Intuitive menu structure and browsing system enable users to easily explore fPoxDB. fPoxDB provides browsing functions, gene distribution table and charts, pre-computed results of eight bioinformatics tools including InterPro scan [21], SignalP 3.0 [45], SecretomeP 1.0f [46], TMHMM 2.0c [47], TargetP 1.1b [48], PSortII [49], ChloroP 1.1 [50], and predictNLS [51], as well as job submission forms for BLAST [41], HMMER [31], BLASTMatrix [32], and ClustalW [42] (Figure 3). In addition, the sequence profiles which were used in prediction of putative peroxidase genes can be downloaded, enabling large scale analysis such as whole proteome search on local computers.

“Browse by Species” displays species name, taxonomy, and the number of predicted peroxidase genes/gene families. For each species, the detail page shows the number of predicted genes for each gene family as a graphical chart and table to present an overview on the peroxidase composition in a genome. The hierarchy implemented in the browser is easy to follow, so that users can readily retrieve data. “Browse by Species” also provides the taxonomically ordered summary table for every peroxidase family where kingdom-level and subphylum-level distribution are available. A summary of the whole database that describes the number of predicted genes against each genome can be downloaded as .csv format. This could provide the possibility to study gene family expansion or contraction across a number of genomes. “Browse by Classes” lists the peroxidase gene families and the number of genes and genomes corresponding to each gene family. Distribution of genes for each gene family is depicted in a box plot in order to show subphylum-level of taxonomic distribution at a glance. These distribution summaries could be used for searching peroxidase families which are limited to a certain range of taxonomy, such as LiP and MnP.

In order to systematically manage the sequence data, fPoxDB website is equipped with “Favorite Browser”, a virtual personal storage and data analysis hub originally developed for CFGP 2.0 (http://cfgp.snu.ac.kr/) [32]. In the “My Data” menu, users can create and manage their own data collections which are synchronized with the CFGP 2.0. The “Favorite” folders and their contents can also be used in the CFGP 2.0 as well as many other family web systems [39,52-54] for further analysis options. For example, the FSD [39] could be jointly used to check how many peroxidases in a Favorite are predicted to be secretory. Furthermore, users can also try 27 bioinformatics tools available at the CFGP 2.0 [32] in the same way. Via the Favorite Browser in fPoxDB, users can submit BLAST [41], HMMER [31], BLASTMatrix [32], and ClustalW [42] jobs with the sequences saved in a Favorite. BLASTMatrix [32] is a parallel BLAST search program which enables searching multiple queries against multiple genomes. The BLASTMatrix [32] offers a wide taxonomic distribution of the query sequences with various viewing options. Users can browse i) gradient aided taxonomic distribution, ii) actual E-value/bit score matrix, and iii) taxonomic conservation of the query sequences. This also enables users to mine putative orthologues in other genomes, which can be stored into a Favorite on the fly. In addition, domain browsing function is available in the Favorite Browser that provides graphical diagrams for selected domains. The image files of domain structures for the sequences in a Favorite can also be downloaded as a zip archive for further use. fPoxDB also has a novel function for investigation of trans-membrane helices (TMHs). By using “Distribution of TMHs” function in the Favorite Browser, position information and sequences corresponding to THM regions, predicted by TMHMM2.0 [55], can be retrieved as a text file. This function may offer starting material for studying structural features or evolutionary relationship of Nox genes as they are known to have conserved histidine residues in their THMs [56,57]. Multiple sequence alignment by ClustalW [42] is also available via the Favorite Browser. Since many protein domains found in peroxidases are highly conserved, site-directed mutagenesis of conserved catalytic residues had been a vibrant research field [12,13,58-61]. Users can align their sequences in a Favorite as full length or a domain of choice, enabling targeted investigation on catalytic domains.

## Conclusions

fPoxDB is a fungi-oriented database for studying comparative and evolutionary genomics of various peroxidase gene families. This database provides more accurate prediction of genes encoding Nox and NoxR in fungi. The web interface of fPoxDB provides i) browsing by species/gene family, ii) kingdom-/subphylum-level of distribution, iii) similarity search tools (BLAST [41], HMMER [31], and BLASTMatrix [32]), iv) multiple sequence alignment by ClustalW [42], and v) domain and TMH analysis function via Favorite Browser. By taking full advantage of these functionalities, fPoxDB will be a valuable platform in i) preparation of data sets for evolutionary study, ii) finding candidate catalytic residues from domain alignment, and iii) finding possible orthologues in other genomes from BLASTMatrix [32] results. In order to provide better prediction and usability, this database will be updated with continuous improvement on gene family definitions, additional fungal genome sequences, and installation of useful analysis functions. Collectively, fPoxDB will serve as a fungi-specialized peroxidase resource for comparative and evolutionary genomics.

## Availability and requirements

All data and functions described in this paper can be freely accessed through fPoxDB website at http://peroxidase.riceblast.snu.ac.kr/ via the latest versions of web browsers, such as Google Chrome, Mozilla Firefox, Microsoft Internet Explorer (9 or higher), and Apple Safari. The data sets supporting the results of this article are included within the article and its additional files.

## Competing interests

The authors declare that they have no competing interests.

## Authors' contributions

JC and YHL designed this project. JC and ND developed the pipeline. JC developed the database and web interfaces. JC, ND, and KTK conducted data analysis. JC, ND, KTK, FOA, JPTV, and YHL wrote the manuscript. All the authors read and approved the final manuscript.

## Supplementary Material

Additional file 1 **Summary table of the number of genes encoding peroxidase gene families in 216 genomes from fungi and Oomycetes.** The summary table shows a taxonomically ordered list of 216 genomes with the number of genes belonging to each peroxidase gene family. Click here for file

Additional file 2 **Reconciled species tree of catalases.** The reconciled tree of catalases from 32 species covering fungi, Oomycetes, animals and plants was constructed. In order to construct a gene tree based on domain regions, catalase domain (IPR020835) was retrieved from the 109 protein sequences. Multiple sequence alignments and construction of a phylogenetic tree was performed by using T-Coffee [30]. A species tree was constructed using CVTree (version 4.2.1) [62] with whole proteome sequences with K-tuple length of seven. The number of duplication and loss were inferred from the reconciliation analysis conducted by Notung (version 2.6) [63] with the catalase domain tree and whole proteome phylogeny. The numbers of gene duplication (D), conditional duplication (cD) and loss (L) events are condensed to the species tree and shown in the corresponding internal node. The number of catalase genes, the species name and the species-level of events are presented next to the leaf nodes. Species names are abbreviated as the following: Fg (*Fusarium graminearum*), Fo (*Fusarium oxysporum*), Cg (*Colletotrichum graminicola* M1.001), Mo (*Magnaporthe oryzae* 70*–*15), Pa (*Podospora anserina*), Nc (*Neurospora crassa*), Bc (*Botrytis cinerea*), Bg (*Blumeria graminis*), Mg (*Mycosphaerella graminicola*), Hc (*Histoplasma capsulatum* H88), Ci (*Coccidioides immitis*), Af (*Aspergillus fumigatus* Af293), An (*Aspergillus nidulans*), Sp (*Schizosaccharomyces pombe*), Sc (*Saccharomyces cerevisiae* S288C), Ca (*Candida albicans*), Mlp (*Melampsora laricis*-*populina*), Pg (*Puccinia graminis*), Cn (*Cryptococcus neoformans* var. *grubii* H99), Lb (*Laccaria bicolor*), Pc (*Phanerochaete chrysosporium*), Hi (*Heterobasidion irregulare* TC 32–1), Sl (*Serpula lacrymans*), Bd (*Batrachochytrium dendrobatidis* JAM81), Pb (*Phycomyces blakesleeanus*), Ro (*Rhizopus oryzae*), Pi (*Phytophthora infestans*), At (*Arabidopsis thaliana*), Os (*Oryza sativa*), Ce (*Caenorhabditis elegans*), Dm (*Drosophila melanogaster*) and Hs (*Homo sapiens*). Click here for file
