# Supplementary figures and images for: fPoxDB: fungal peroxidase database for comparative genomics
Source: BMC Microbiol. 2014 May 8;14:117. doi: 10.1186/1471-2180-14-117 (PMC4029949; doi:10.1186/1471-2180-14-117)

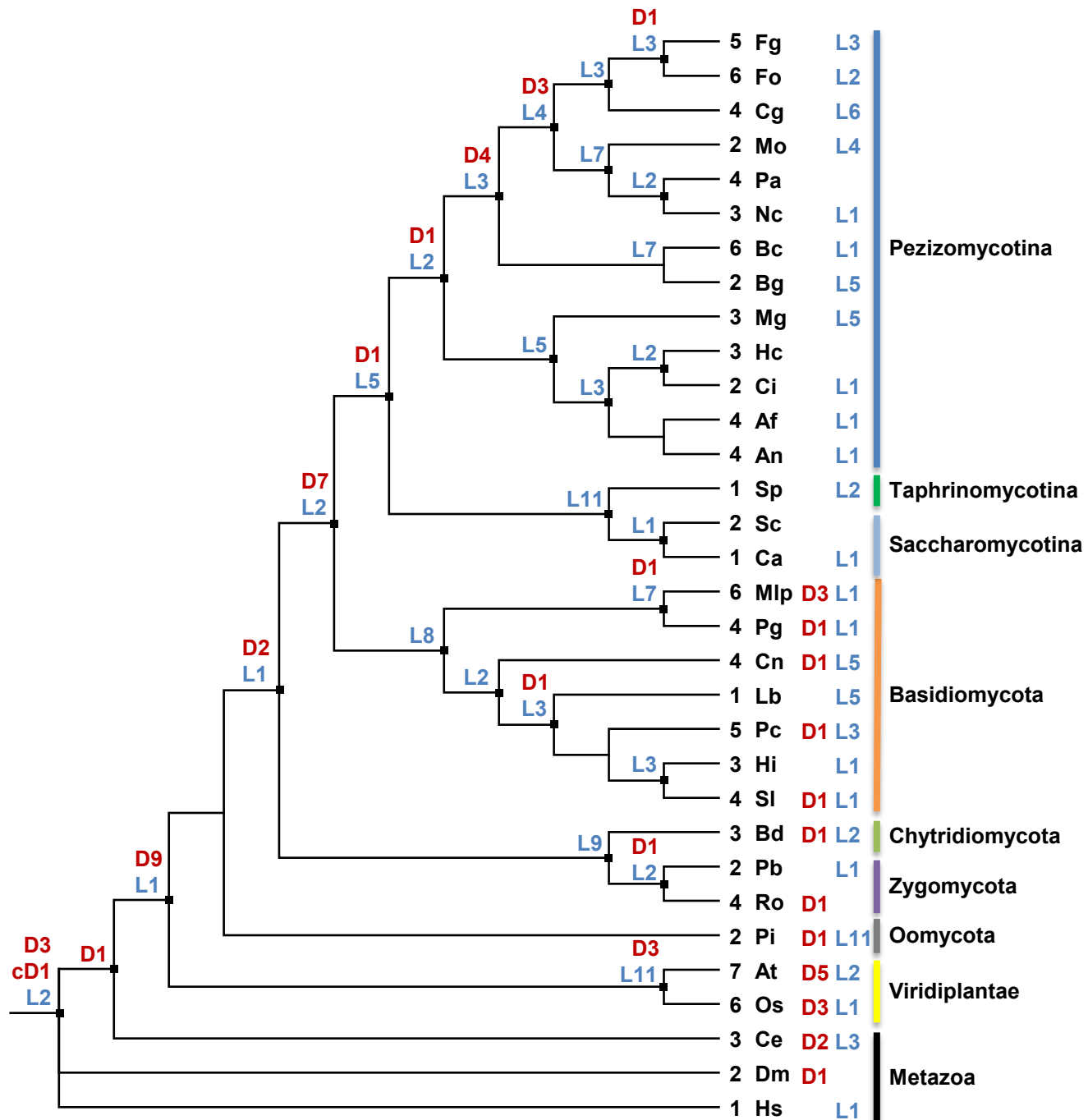

Supplement: Additional file 2 — Reconciled species tree of catalases. The reconciled tree of catalases from 32 species covering fungi, Oomycetes, animals and plants was constructed. In order to construct a gene tree based on domain regions, catalase domain (IPR020835) was retrieved from the 109 protein sequences. Multiple sequence alignments and construction of a phylogenetic tree was performed by using T-Coffee [30]. A species tree was constructed using CVTree (version 4.2.1) [62] with whole proteome sequences with K-tuple length of seven. The number of duplication and loss were inferred from the reconciliation analysis conducted by Notung (version 2.6) [63] with the catalase domain tree and whole proteome phylogeny. The numbers of gene duplication (D), conditional duplication (cD) and loss (L) events are condensed to the species tree and shown in the corresponding internal node. The number of catalase genes, the species name and the species-level of events are presented next to the leaf nodes. Species names are abbreviated as the following: Fg (Fusarium graminearum), Fo (Fusarium oxysporum), Cg (Colletotrichum graminicola M1.001), Mo (Magnaporthe oryzae 70–15), Pa (Podospora anserina), Nc (Neurospora crassa), Bc (Botrytis cinerea), Bg (Blumeria graminis), Mg (Mycosphaerella graminicola), Hc (Histoplasma capsulatum H88), Ci (Coccidioides immitis), Af (Aspergillus fumigatus Af293), An (Aspergillus nidulans), Sp (Schizosaccharomyces pombe), Sc (Saccharomyces cerevisiae S288C), Ca (Candida albicans), Mlp (Melampsora laricis-populina), Pg (Puccinia graminis), Cn (Cryptococcus neoformans var. grubii H99), Lb (Laccaria bicolor), Pc (Phanerochaete chrysosporium), Hi (Heterobasidion irregulare TC 32–1), Sl (Serpula lacrymans), Bd (Batrachochytrium dendrobatidis JAM81), Pb (Phycomyces blakesleeanus), Ro (Rhizopus oryzae), Pi (Phytophthora infestans), At (Arabidopsis thaliana), Os (Oryza sativa), Ce (Caenorhabditis elegans), Dm (Drosophila melanogaster) and Hs (Homo sapiens). [file 1471-2180-14-117-S2.pdf]
